# On Septicæmia

**Published:** 1877-02

**Authors:** S. Messenger Bradley

**Affiliations:** Surgeon to the Manchester Royal Infirmary


### Selections.

---

ON SEPTICÆMIA.—*By S. Messenger Bradley, Esq., Surgeon to the Manchester Royal Infirmary.*—In June last year I admitted a man into the Manchester Royal Infirmary with hydrocele of the tunica vaginalis, which had resisted ordinary means of cure. The day after admission I tapped him once more, when, for the first time, I discovered a second sac, encysted hydrocele of the cord, on the same side. This I also tapped, and injected both sacs with tincture of iodine. This little operation was followed by suppuration, and on the third day by grave constitutional disturbance; temperature rising to 108°, and the pulse to 130; countenance anxious; tongue dry and furred; much restlessness, profuse sweatings, and rigors.

The sac of the tunica vaginalis was freely laid open and half a pint of fetid pus evacuated. The general symptoms were for a short time relieved; but the following day diarrhœa ensued, the temperature again rose, and the patient died delirious six days after the paracentesis had been performed. There was no evidence of thrombosis, embolism, or visceral abscesses; in a word, the man died of septicæmia.

A few months after this, Walter Searson, curator of the Owens College Medical School, pricked his thumb while dissecting a tiger for the museum, and two days afterwards began to suffer pain at the seat of injury. On the fourth day he called to see me, when I found a small quantity of thin fluid discharging at the root of the thumb-nail. The lymphatics were perceptibly affected, the skin was cool, the tongue dry, pulse 100; but perhaps the most noticeable symptom was intense depression of spirits. I freely incised the thumb and sent him home to bed; this was Aug. 22d. From this day his general condition grew worse, up to the 26th, when an ill-defined, or rather an undefined, abscess appeared in the cellular tissues of the arm. One being opened several ounces of pus were discharged, which lay loosely in the tissues unconfined by any limiting membrane. Each day fresh abscesses of the same diffluent character appeared on the chest, abdomen, legs and feet. He was very restless, and passed gradually from a condition of prostration into one of muttering delirium, which ended in death on September 11th, twenty-four days after the reception of the poison. The secretion of urine was almost entirely suppressed for the twenty-four hours preceding death, and the breathing was both rapid and difficult, auscultation revealing extensive moist crepitation of both lungs. There was no post-mortem, but this was clearly a case of pyæmia.

At the same time that the man with hydrocele lay dying of septicæmia, a youth with a compound fracture of his leg was brought into the same ward. He was a healthy lad, and there was nothing in the case to cause alarm; indeed, it was one of those cases of which, with proper surroundings, we may safely predicate a sure and speedy recovery. After lying however, in juxtaposition to the septicæmic patient, the young fellow fell a victim to the same disease, and it was only after two months of great peril that he contrived to drift through the disease into a slow convalescence.

These three cases may serve as types; the first arising *de novo*, the septic material being manufactured by the patient himself—a consequence only possible in certain conditions of the system. This is the class met with in private practice. The second case illustrates the production of the disease by direct inoculation from without; while the third affords an illustration of the mode in which the malady is conveyed in hospital from patient to patient.

I draw no distinction between septicæmia and pyæmia, believing that if there is a distinction, it is a distinction without a difference. Both are caused by organic matter (septic germs) entering the general system: in the first and third cases entering probably by the veins; in the second by the lymphatics. In the first case the poison killed without visceral lesion; in the second it gave rise to thrombosis in the veins, which, being detached, became the nuclei of suppurations scattered throughout the cellular tissues generally and the various viscera. But clearly these are only unimportant varieties of one pathological condition, which we may conveniently term septicæmia. Now in studying this condition of septicæmia, we shall learn something of its nature and how to prevent it by turning to another variety of the same disease—I mean that form of puerperal fever which depends for its production upon puerperal peritonitis. As in ordinary pyæmia, cases of puerperal fever occur sporadically, so to speak—spring up away from a hospital, in patients surrounded by everything which we should consider antagonistic to its development. In these cases, however, we can nearly always trace the presence of some putrefying organic matter—a bit of placenta, etc.,—just as we can in isolated instances of pyæmia, as in ordinary pyæmia, too, puerperal peritonitis is clearly contagious, conveyed with fatal certainty from one patient to another by the hands of doctor or midwife.

We can, however, advance still further towards a conclusion as to the real character of the poison and as to the mode in which it must be destroyed. The experiments of Chauveau and Burdon Sanderson demonstrate that if a septic fluid, capable of producing toxæmia when injected into the veins of a living animal, be strained through a porcelain filter, the liquor so filtered may now be injected with impunity, whereas the solid residue remaining on the filter retains in full force all the septic properties of the orignal fluid matter. This residue is shown to entirely consist of bacteria. Whether these bacteria be the very incarnation of the poison, or merely act as beasts of burden for it, is a question which exercises the minds of curious men, but need not here detain us, having established the fact that bacteria are essential to the production of septicæmia.

We have learned a good deal of late about these organisms. We know, amongst other things concerning them, that they are heavier than air—e. g., in Tyndall's recent experiments upon the non-septic properties of optically pure air it is shown that three days suffice for gravitation to deposit all the floating germs upon the bottom of the little boxes which he uses; and that the poison of septicæmia is likewise of a comparatively heavy nature is evident in the clinical history of puerperal fever, which teaches us that the spread of the disease may always be traced to human go-betweens, and is never conveyed from patient to patient by mere diffusion through the air. It is of course possible that, in a crowded hospital, poison-germs may be wafted from the wound of a septicæmic patient and alight upon the open sore of a neighboring case, when it will, in turn, give rise to the disease; but the whole history of septicæmia differs markedly from diseases which, in their wide and rapid spread, seem to depend upon the diffusion of septic gases.

Assuming, then, the point to be conceded that septicæmia does not depend on gaseous diffusion, and coupling the facts that it never occurs without putrefying organic matter and that putrefaction requires the presence of living germs, we are not postulating too much in affirming that the destruction of the germs must render the spread of the disease impossible. To what practical issues does this lead us? You all know what just odium attaches to the surgeon who, after one clear case of puerperal fever, allows himself to become the medium of its conveyance to another victim; not only moral pains and penalties are his, but the arm of the law deals with him sharply. Now I confess it seems inconsistent to be so careful in one case and so careless in the other. All authorities admit the contagiousness of pyæmia; nearly all agree that it is carried only by actual contact from case to case; and yet we see, every day, cases of pyæmia remaining in wards crowded with open wounds (wounds with open vessels ready to absorb everything, good or bad), and all these cases under the care of the same surgeon, house-surgeon, and nurses. Surely it is not asking too much to insist upon a separate ward, where any case of septicæmia should be transferred, and which ward should be looked after by an entirely separate staff of attendants.

But it is not enough to isolate our cases; we must purify our wards; and this subject naturally divides itself into two:—1st. What test have we, besides that of the ill-doing of our patients, that our wards are impure? 2nd. What are the best means of puiifying them? Unfortunately chemistry, to which we naturally turn, yields no satisfactiory reply as to the detection of organic impurities. But it does give us an answer, though in a somewhat roundabout way; for, having discovered that the quantity of carbonic acid in the air is constant, being almost exactly the same in a London court and at the summit of Mont Blanc, and that any excess in the carbonic acid is an indication of organic impurities in the air,—in this way chemistry does enable us to get at some precise results. Let us, then, adopt this method of investigation in examining the condition of our own hospital. In accordance with my request, Mr. Thomas Harrison, F. C. S., a very competent authority, on January 14th of this year, examined the air of certain wards in the hospital with the following results:

*Estimate of the quantity of* $CO_2$ *in 10,00 volumes of air.*

Average quantity $CO_2$ in 10,000 volumes of Manchester air, 4.

| | |
|---|---|
| President ward .. .. .. .. .. | 18 |
| Humphry Nicholl ward .. .. .. | 8 |
| Passage adjoining .. .. .. .. | 4 |

I shall not further allude to the condition of the President ward than to say that, be the case what it may, such a condition of air as is here revealed is an unfit atmosphere for sick people. What I wish more particularly to draw your attention to is the condition of Humphry Nicholl ward. Owing to a recent epidemic of erysipelas, this ward had been cleared out a month before the analysis was made, and both windows and ventilators kept open ever since; yet we find that the quantity of carbonic acid is exactly double that in the adjoining passage. If you ask me how I explain this remarkable fact, I can only answer that I imagine it to be caused by hordes of bacteria which are for ever discharging carbonic acid into the air like so many little Christians. This supposition, which I quite admit seems somewhat fanciful, receives support from another experiment performed by Mr. Harrison, who, on January 16th, examined the air of the ashpit of his back yard. On this occasion he found 14-08 parts of carbonic acid in 10,000 volumes of air. Having ascertained so much, he discharged a quantity of ozone for thirty seconds, and then found only 10.2 parts of carbonic acid. Now, as ozone has no effect upon carbonic acid, this result is only explicable on the theory that ozone arrests the fresh formation of carbonic acid. We know that ozone destroys the life of bacteria; we know also that bacteria emit carbonic acid: hence it seems a fair inference that the extraordinary quantity of carbonic acid is due to the presence of aerial hosts of those minute organisms.

That bacteria do exist in the air of our hospital is quite certain. In 1868 Mr. Lund examined the air of the hospital, and found bacteria in abundance. Dr. Dreschfield, at my request, lately exposed some papers moistened with water and glycerine to the air of some of the hospital wards, and found bacteria, though, he informs me, in very small numbers. I supplemented his experiments by repeating those which Mr. Lund had made, and found that the distilled water with which the flasks I used were filled became turbid with bacteria in a few days. Now, bearing in mind the fact that ozone destroys organic matter, and remembering that it is organic matter which gives rise to the putrefactive changes of septicæmia, let us inquire how we can practically bring this agent to bear. I find that the air of Manchester is absolutely devoid of this natural antiseptic, but it fortunately happens that nothing is easier than to manufacture ozone, and this, too, quite on a grand scale. With a Rumkopf coil, such as this, and one of these generators of Tisley's, you could fill the largest ward in this hospital with ozone in a few hours. Now that I put the battery in action, so as to electrify the oxygen as it passes through the tube, you see the gas in its changed state coming off in large quantities, blackening this prepared paper, and changing this solution of starch and iodide of potassium into a deep and beautiful blue. Without venturing to affirm that every antiseptic acts simply as an oxidising agent, we may safely affirm that no aniseptic is so potent as nascent oxygen or ozone; hence it seems to me that when an epidemic of septicæmia has vitiated the air of our wards we should resort to this agent for purification.

These experiments, to which I have before alluded, may be taken as proving that the air of the hospital is impure; yet, if we examine our statistics, we find that they compare favorably with those of most other hospitals. The best cases to take are probably amputations, as they are numerous enough to afford reliable grounds for comparison, and do not depend for their result on any special skill in the operator. Professor Simpson, whose *hospital* statistics were never impugned, stated that "all amputations of all the four limbs in eleven of the largest general hospitals of the country gave a mortality of 41.6 per cent., or nearly 1 in 24." Erichsen found that 80 consecutive cases of amputations at University College Hospital, from July 1, 1870, to December 1, 1873, gave a mortality of 26.2 per cent. Of 307 amputations performed by himself, 79, or 25 per cent., died. At our own hospital, from 1868 to 1873, 304 amputations, whose results are tabulated, were performed, excluding all amputations of the hand and lower third or forearm and of the foot and ankle. Of these 77 died, giving a mortality of 25 per cent.; if, however, we include the previously excluded amputations, we reduce the mortality from 25 to 20 per cent., being 2 per cent. below that of University College Hospital, and 2 per cent. above that of St. Bartholomew's, where the mortality of late years has been reduced to 18 per cent. But, although by cleanliness and care we have reduced the mortality to a percentage which compares favorably with that of the best metropolitan hospitals, we should not rest satisfied with such a result when we are acquainted with the fact that in a bygone generation, and with ruder means at his command, Guthrie, during the Peninsular war, lost but one case in twelve of all his amputations, being a mortality of only 8 per cent. We should not, indeed, justly rest satisfied until we have achieved an equal success with his, especially when we know the only cause for the great disparity lies in the comparative purity of the air. At the debate on pyæmia at the Clinical Society last year, Spencer Wells and Dr. Gordon made statements bearing upon this question, the one referring to the hospitals at Malta, the other to the Parisian ambulance during the Franco-Prussian war; their statements prove that the mortality was ever in direct proportion to the quality and quantity of the air.

As I have said, there is no ozone in the air of our city, but it is abundantly present in the outskirts, and these papers, which are so deeply colored by ozone, received the tinge by being exposed for a few hours in that of Oxford-road, where the Infirmary Committee urge the erection of the future hospital.

To what conclusions do these thoughts led us? Let me briefly relate the case by way of answer. I have endeavored to show that pyæmia, septicæmia, and puerperal peritonitis (all varieties of one disease, which may be generically termed septicæmia) are associated with the development of organic germs, "bacteria," which act either as the carriers of the poison or as the poison itself; that septicæmia may be, and is, carried in a hospital from one patient to another by surgeon or nurse; that all cases of septicæmia occurring in a hospital should be at once removed and effectually isolated from contact with the other cases; the ozone destroys the vitality of bacteria, ozone should be employed to purify our wards when septicæmia has appeared in them; that this may be readily effected in a few hours by means of a small battery and Tisley's ozone generator; that the air in the Manchester Infirmary is impure, and therefore deleterious, but that, notwithstanding this, our death-rate, in consequence probably of the great attention paid to cleanliness, compares favourably with that of other hospitals; that, however, when we recall the achievements of Guthrie in the Peninsular war of sixty years ago, we must consider this mortality really excessive, and should constantly urge the necessity of our hospital being built on better principles and in a purer air.—*Lancet, May* 27, 1876, *p.* 768.

---

Absence of the Uterus, with a Previous History of Chronic Inversion of this Organ, which was mistaken for Polypus, and Removed by Ligature.—*By W. R. Whitehead, M.D., of Denver, Colorado.*—The removal of the uterus is of sufficient interest, under any circumstances, to merit grave consideration; but when the result of an error of diagnosis, every physician will

